# Discrepancies between radiological and histological findings in preoperative core needle (CNB) and vacuum-assisted (VAB) breast biopsies

**DOI:** 10.1007/s00432-020-03481-7

**Published:** 2020-12-07

**Authors:** Inna Jörg, Jann Wieler, Constanze Elfgen, Kristina Bolten, Claudia Hutzli, Julia Talimi, Denise Vorburger, Matthias Choschzick, Linda Moskovszky, Konstantin Dedes, Zsuzsanna Varga

**Affiliations:** 1 Department of Gynecology, Hospital Triemli, Zurich, Switzerland; 2 Institute of Diagnostic Radiology, University Hospital Zurich, Zurich, Switzerland; 3 Breast Center Seefeld, Zurich, Switzerland; 4 Department of Radiology, University Witten-Herdecke, Witten, Germany; 5 Department of Gynecology, Hospital Zollikerberg, Zollikerberg, Switzerland; 6 Department of Gynecology, Hospital Männedorf, Zurich, Switzerland; 7 Breast Center, University Hospital Zurich, Zurich, Switzerland; 8 Comprehensive Cancer Center Zurich, University Hospital Zurich, Zurich, Switzerland; 9 Institute of Pathology and Molecular Pathology, University Hospital Zurich, Schmelzbergstrasse 12, 8091 Zurich, Switzerland

**Keywords:** Breast biopsy, Discrepancy, Imaging, Histology

## Abstract

**Background:**

Ultrasound (US)-guided breast biopsy is a routine diagnostic method used to correlate imaging finding to a histological diagnosis which is still the gold standard in preoperative diagnostics. The accuracy of US-guided breast biopsies relies on a precise radiologic-histopathologic correlation, which is discussed amongst an interdisciplinary team of gynecologists, radiologists and pathologists. However, false-negative or non-diagnostic biopsy results occur. Hence, a thorough and honest discussion to clarify the reason for discrepancies and to decide the next diagnostic step between specialists of the different disciplines is warranted. In this retrospective study, we analyzed discrepant findings between imaging and pathology results on preoperative breast biopsies.

**Methods:**

Core and vacuum-assisted breast biopsies from 232 patients were included in this study. Inclusion criteria were (1) non-diagnostic (B1) category on histology independent from imaging category and (2) histological benign (B2) category with a BIRADS 5 (Breast Imaging Reporting and Data System) rating on imaging. Histological diagnoses were retrieved from all cases. Follow-up data were available in most cases.

**Results:**

138 biopsies were classified as B1, 94 biopsies as B2 category. 51 of 138 B1 cases (37%) underwent re-biopsy. Re-biopsy found malignancy (B5) in 19 of 51 cases, and B3/4 (premalignant) lesions in 3 of 51 cases. All B2 cases underwent second-look imaging-diagnosis, in 57 of 94 cases (66%) consecutive direct surgery or re-biopsy. Of these, malignancy was diagnosed histologically in 26 of 57 cases (45.6%).

**Conclusion:**

Determining imaging-pathology concordance after US-guided breast biopsy is essential. Discrepant cases and further diagnostic steps need to be discussed with an interdisciplinary approach.

## Introduction

US-guided biopsy on palpable and non-palpable breast lesions is a routine diagnostic tool in the preoperative setting. Core needle biopsy (CNB) is less invasive than vacuum-assisted biopsy (VAB) and therefore fewer surgical complications are expected (Mihalik et al. 2010). In the case of benign findings in the biopsy, unnecessary surgery can be avoided. In addition, the cost of a biopsy is lower than that of an open surgical excision (Kim et al. 2008). In case of malignant findings, weekly preoperative senology boards provide recommendations on the basis of which individual options can then be discussed with a patient. These also depend on age, tumor stage, receptor status and of course, the patient’s wishes.

The key of a successful core biopsy workflow is an excellent communication and a high level of agreement among attending radiologists, pathologists and gynecologists (Hahn et al. 2012; Parikh and Tickmann 2005). The breast sonographer is the most experienced in evaluation of abnormal findings, thus playing an essential role in the diagnostic process. Preferably, the same person who detected the lesion concurrently performs the biopsy to ensure accurate analysis of the lesion. This continuity helps to ensure the lesion recommended for biopsy is the one that actually undergoes biopsy as well as a direct assessment of its technical adequacy. The pathologist is crucial in assessing and communicating the quantitative and qualitative aspects of the biopsy. Literature shows the frequency of missed cancers in trials ranges from 0.3 to 8.2%, with approximately 70% being identified immediately after core biopsy. Thirty percent are delayed false-negative stereotactic breast biopsies where a further biopsy is needed in 9–18% of patients (Mihalik et al. 2010).

Since the 2003 edition of the BIRADS was published, the BIRADS lexicon and classification have proven to be useful in predicting the likelihood of malignancy in radiologically assessed breast lesions (Kim et al. 2008; Lee et al. 2008; Park et al. 2018). Each BIRADS assessment category indicates an anticipated likelihood of malignancy, which is based on a thorough evaluation of the imaging features Kim et al. 2008, Lee et al. 2008.

Category 3 (probably benign) is reserved for specific imaging findings known to have a likelihood of malignancy of > 0% but ≤ 2%. Such lesions include solid masses with a circumscribed margin, oval shape and parallel orientation; complicated cysts and clustered microcysts (Mendelson et al. 2013). Although the recommended management is imaging follow-up, CNB can be performed under certain circumstances. For category 3 lesions, a benign (B2) biopsy result can be regarded as concordant with the images. Malignant biopsy results (B5) are considered to be discordant, but the sonographic features should be reviewed for subtle suspicious imaging features that might have been overlooked in the first place (Youk et al. 2011).

Category 4 (suspicious of malignancy) covers a wide range of likelihood of malignancy, ranging from 2 to 95%. Thus, almost all recommendations for image-guided breast interventions come from assessments made using this category. Starting in the fourth edition of BIRADS and maintained in its recent fifth edition, category 4 is subdivided into 4A, 4B, and 4C (Mendelson et al. 2013). The range of the likelihood of malignancy is > 2% and ≤ 10% for category 4A, > 10% and ≤ 50% for category 4B, and > 50% and < 95% for category 4C. Although the subcategorization of sonographic BIRADS category 4 has been reported to be useful in predicting the likelihood of malignancy, established objective criteria do not exist for the subcategories and inter-observer agreement has been shown to be poor (Mihalik et al. 2010; Youk et al. 2010).

Category 5 (highly suggestive of malignancy) carries a very high probability (≥ 95%) of malignancy, and any benign percutaneous tissue diagnosis should be considered discordant.

The histopathological B classification is defined in an analogous way, such as B1 (not diagnostic), B2 (benign), B3 (lesions with uncertain potential), B4 (suspicious of malignancy) and B5 (malignant) (AGO (2020); S3 Leitlinien 2020; Breast Cancer Screening Program NSH 2020).

In this study, we systematically analyzed discrepant diagnoses between radiological and histological findings, focusing on non-diagnostic biopsies (in all BIRADS categories) and on malignant imaging diagnosis (BIRADS 5) with histologically benign lesions (B2 lesions).

## Materials and methods

### Patient cohort

In total, 232 patients were included in this study. The cohort was retrieved from diagnostic files from the Institute of Pathology and Molecular Pathology, University Hospital Zurich, where between 2010 and 2019 more than 10,000 diagnostic breast biopsies were performed.

The study was covered by a valid ethical approval (Zurich Cantonal Ethical Committee, KEK–2012–554 including regulation on informed consents).

Following groups were analyzed:

First group: Patients who were biopsied because of radiological lesions classified from BIRADS 2 to BIRADS 5, where a B1 lesion was reported in the histology.

Second group: Patients who were biopsied because of radiological lesions classified as BIRADS 5 where a B2 lesion was diagnosed in the histology.

## Results

### B1 histological findings and any BIRADS category

This group encompassed 138 cases. Patients underwent breast core biopsies because of radiological findings from BIRADS 2 to BIRADS 5 lesion and histological results that showed non-diagnostic B1 lesions.

**Fig. 1:**
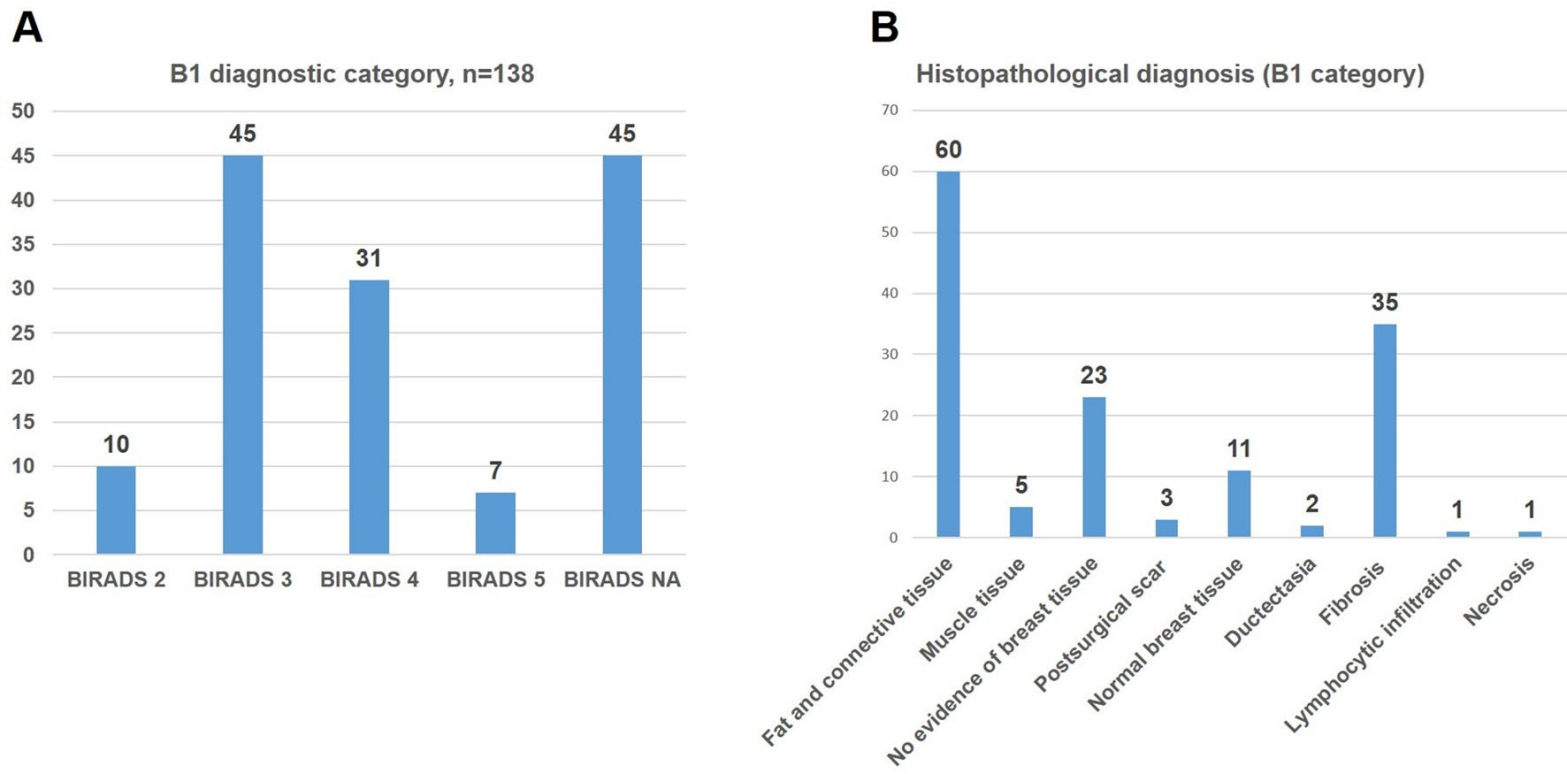
The following diagnostic imaging (**a**) and histological (**b**) diagnoses were found in the primary biopsy with B1 diagnostic category

Ten biopsies were carried out for BIRADS 2 lesion, 45 biopsies for BIRADS 3, 31 biopsies for BIRADS 4 and 7 biopsies for BIRADS 5 imaging categories. In 45 patients, the radiological findings were suspicious for malignancy without documented BIRADS classification (Fig. 1a).

The BIRADS category prior to the US-guided CNB was established by ultrasound diagnostics (in 129 cases), ultrasound in addition to mammography in 7 cases, and ultrasound in addition to MRI in 2 cases. The initial biopsy was always US-guided CNB with 3–5 cores.

Histological findings showed fat/connective tissue in 60 cases (43%) followed by skeletal muscles (5 cases), scar tissue (3 cases), normal breast tissue (11 cases), ductectasia (2 cases), fibrosis (35 cases), inflammation (1 case) and necrosis (1 case). In 23 cases, there was no evidence of breast tissue in the biopsy (Fig. 1b).

**Fig. 2:**
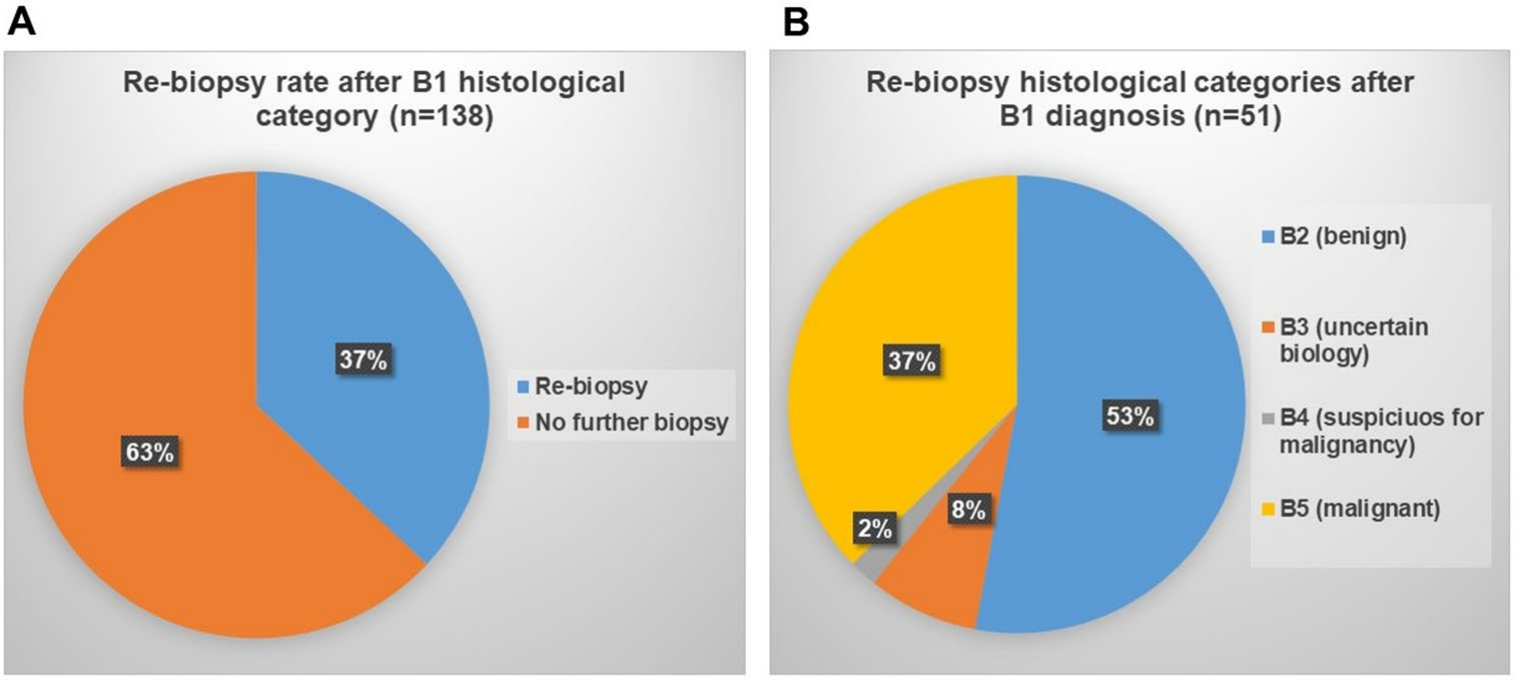
Re-biopsy rate after B1 histological category (**a**) and distributions of B-category in re-biopsies after B1 diagnostic category (**b**)

51 out of 138 patients (37%) underwent re-biopsy (Fig. 2a).

Out of the 51 cases which were re-biopsied, 19 (37%) were diagnosed as B5, one (2%) as B4, 4 (8%) as B3 and 27 (53%) as benign lesions (Table 1, Fig. 2b).

**Table 1:** Histological diagnoses in re-biopsies after B1 category

Re-biopsy category (after B1 diagnosis)	*n*	Histology
B2	27 (53%)	Benign histology
B3	4 (8%)	Classical lobular neoplasia (LN) (*n* = 2) Flat epithelial atypia (FEA) (*n* = 2)
B4	1 (2%)	Atypical ductal hyperplasia (ADH)
B5	19 (37%)	DCIS (*n* = 3) Solid papillary carcinoma (*n* = 1) NST carcinoma (*n* = 11) Lobular carcinoma (*n* = 4)

### Histological B2 category and radiological BIRADS 5 lesions

**Fig. 3:**
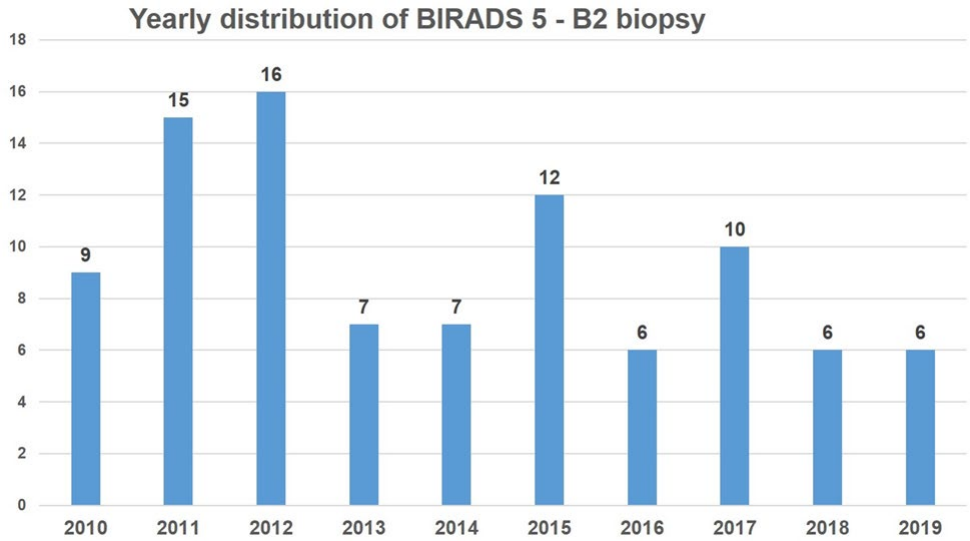
Yearly distribution of BIRADS 5—B2 biopsy categories over a 10-year period

This group consisted of patients with BIRADS 5 in imaging and a B2 diagnosis in histology. We identified 94 biopsies matching this category. The distribution of discrepancies between BIRADS 5 and histologically benign lesions varied from 6 to 16 cases per year (Fig. 3). In more than half of these cases (55%), mastopathic changes were identified: fibrosis in 39 of 94 patients (36%) and adenosis in 18 of 94 cases (19%). Other frequent diagnoses included fibroadenoma, fat tissue necrosis, chronic inflammation and scar formation in the remaining 45% of B2 biopsies (Table 2). The BIRADS category prior to the US-guided CNB was established on ultrasound in 83 cases, ultrasound in addition to mammography in 9 cases, ultrasound in addition to MRI in 1 case and ultrasound in addition to PET CT in 1 further case. Initial biopsy was always US-guided CNB with 4–5 cores.

**Table 2:** Histological diagnoses in the discrepant categories BIRADS 5 and B2 benign histology (*n* = 94)

Scar/scarred fibrosis	3
Adenosis	18
Fibrosis	39
Chronic inflammation	7
Fibroadenoma	10
Fatty necrosis	7
Mastopathic changes, usual ductal hyperplasia (UDH)	10

**Fig. 4:**
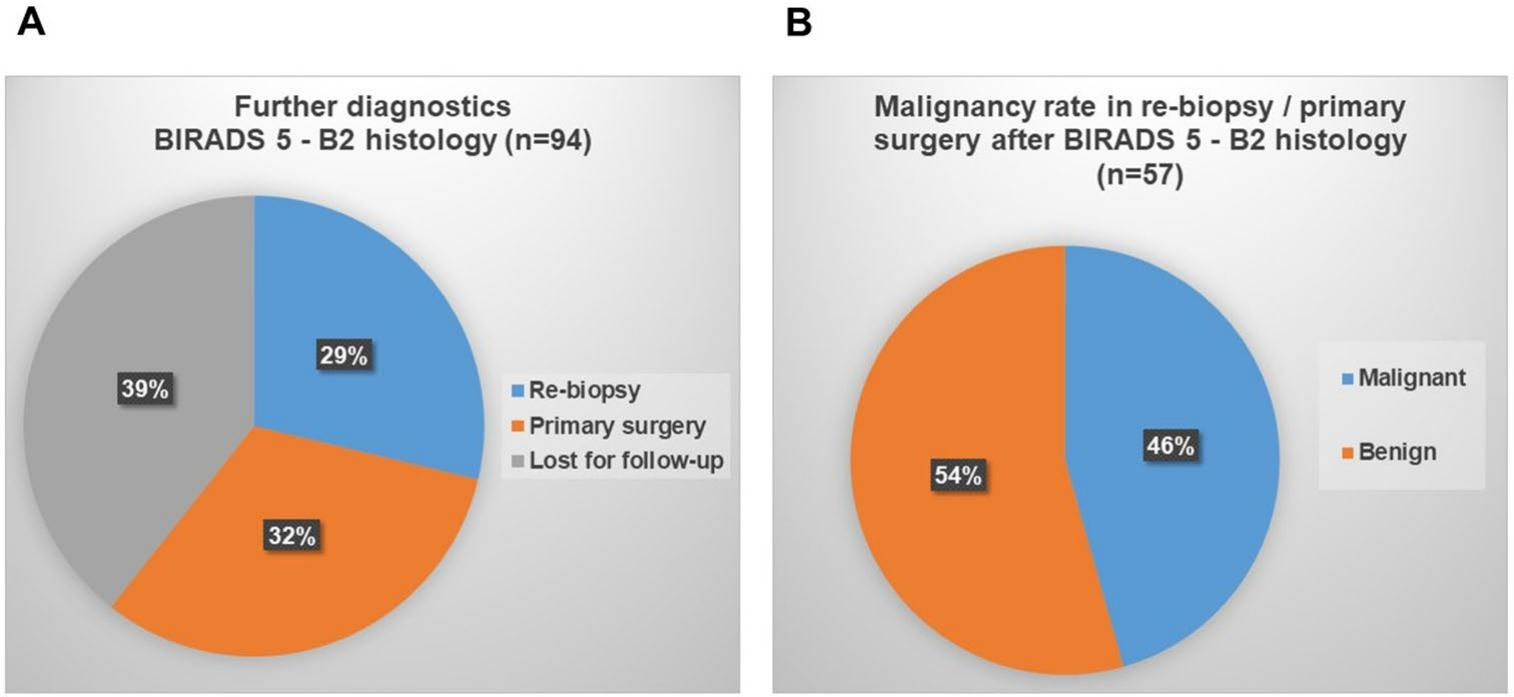
Repeat diagnostic procedure after benign (B2) histology at BIRADS 5 imaging category (**a**) and malignancy rate in re-biopsies/primary surgery after benign (B2) histology at BIRADS 5 imaging category (**b**)

In 57 of the 94 cases, further diagnostics were carried out including a radiology-pathology correlation, US and in some cases MRI (60.6% of all BIRADS 5 cases). Interestingly, from 2010 to 2012, radiologically suspicious but histologically benign (B2) lesions most frequently were handled by open surgery, while from 2012 to 2016, a re-biopsy with CNB was performed, and from 2017 to 2019, VAB increasingly was used for discordant findings (Fig. 4a). Of the 57 cases, which were followed up by further diagnostics as described above, malignancy was confirmed histologically in 26 cases (45.6%) (Fig. 4b).

## Discussion

In this study, we analyzed cases in which a discordance between radiological (probably malignant or malignant) and histological (benign) findings was observed. A case was included when imaging features of a lesion that were suspicious for malignancy (i.e., BIRADS category 4 or 5), corresponded to benign histology (B2 category) in the CNB. The reported percentages of imaging-pathology discordant lesions among breast CNB range from 2.0 to 19.2% (Soyder et al. 2015; Sohn et al. 2014; Youk et al. 2010; Son et al. 2011). Histologically benign lesions with speculated findings, such as granular cell tumor, sclerosing adenosis, post-surgical scar, fatty necrosis, mastitis, diabetic mastopathy and sarcoidosis, can mimic malignancy on US (Cho and Park 2013; Kim et al. 2007). In our cohort, approximately half of BIRADS-5 cases turned out to be malignant in re-biopsy or in open surgery, and the other half were confirmed benign.

The histopathological B classification is defined in several classification systems, such as the NHS Breast Cancer Screening program, the AGO guidelines, the S3 guidelines and also by the Minimal Invasive Breast Biopsy (MIBB) classification (AGO (2020); S3 Leitlinien 2020; Breast Cancer Screening Program NSH 2020). Category B1 is defined as normal tissue, lack of orientating breast parenchyma or insufficient tissue. Category B2 applies to benign lesions (such as fibroadenoma, usual ductal hyperplasia, fibrosis and cysts, among others). Category B3 encompasses biologically uncertain lesions (such as flat epithelial atypia, atypical ductal hyperplasia, classical lobular neoplasia, radial scar and benign/borderline phyllodes tumors). Category B4 is usually applied when a DCIS (or at least an ADH) is suspected, and category B5 describes malignancy (AGO 2020; S3 Leitlinien 2020).

The question of whether the BIRADS 5 category on ultrasound and/or mammography scans is correctly applied or is set too high for discrepant lesions, can only be answered by directly correlating the histological and the imaging findings in each given case (Parikh and Tickmann 2005; Son et al. 2011; Park et al. 2018).

Category B1 with normal breast tissue may indicate that the target lesion was not reached by the US-guided CNB. The documentation of the performance of the biopsy with regard to the US image (an ultrasound picture of the biopsy needle within the target lesion) is therefore of utmost importance. Sufficient experience in US-guided CNB has been shown to decrease the rate of misguided target lesions (Li et al. 2010; Mihalik et al. 2010). Especially in discordant cases, the size of the CNB should be considered as thinner needles may potentially contribute to missed lesions (Li et al. 2010; Mihalik et al. 2010).

Sonographic findings in special situations, such as scarring—as well as post-operative and post-irradiation connective tissue changes—may pose an even more difficult imaging interpretation, especially in view of the differential diagnosis of a recurrent lesion (Park et al. 2018). In discrepant cases, it is also essential for pathologists to re-view B1 and B2 diagnostic categories on the histological slides.

The interdisciplinary direct communication and feedback between the radiologist/gynecologist and pathologist is of utmost importance to enhance the learning effect and to clarify the reason for discrepant findings (Park et al. 2018; Mihalik et al. 2010; Soyder et al. 2015; Sohn et al. 2014; Youk et al. 2010).

In our study, more than half of initially discordant lesions that were biopsied with US-guided CNB were confirmed be malignant in subsequent open excision, VAB or re-biopsy (Mihalik et al. 2010; Soyder et al. 2015; Sohn et al. 2014; Youk et al. 2010). Discordant benign histological findings need to be immediately discussed between the performing radiologist/senologists and the interpreting pathologist, ideally even within an interdisciplinary preoperative senological board to clarify the discrepancy. Based on the decision of the multidisciplinary board, the radiologist should communicate with the referring physician as well as with the patient and discuss the need—or the lack thereof—for a repeated biopsy (Mihalik et al. 2010; Soyder et al. 2015; Sohn et al. 2014; Youk et al. 2010; Park et al. 2018; Parikh and Tickmann 2005).

In addition to CNB, a US-guided VAB has been shown to be a valuable alternative to open surgery for breast lesions with imaging-pathology discordance with an upgrade rate from 4.6 to 22.7% (Kim et al. 2012; Li et al. 2010; Wang et al. 2011). Therefore, both CNB and US-guided VAB can be an option for repeated biopsies of discordant benign lesions. The best biopsy method should be determined individually after consultation among the radiologists, pathologists, referring physicians and the patient.

## Conclusion

US-guided breast CNB is an accurate method for diagnosing breast cancer, with a false-negative rate ranging from 0.1 to 2.5%. Determining imaging-pathology concordance after US-guided breast CNB is essential and needs to be addressed within an interdisciplinary preoperative conference. Discrepant findings should undergo a second-look review of imaging and histological findings, and the next consecutive diagnostic step should be decided within an interdisciplinary team. Appropriate management, including active communication between the pathologist, referring physician and patient, should be carried out accordingly.

## Data Availability

All data are available upon request without restriction.
